# Clinical and Diagnostic Features of Feline Epilepsy: Distribution of Seizure Types and Associated Factors

**DOI:** 10.3390/ani15233497

**Published:** 2025-12-04

**Authors:** Martinas Jankauskas, Julija Tamosauskaite, Aistė Gradeckienė, Sigitas Čižinauskas, Dmitrij Kvitka, Vita Riškevičienė

**Affiliations:** 1 Veterinary Academy, Lithuanian University of Health Sciences, 44307 Kaunas, Lithuania; aiste.gradeckiene@lsmu.lt (A.G.); sigitas.cizinauskas@aisti.info (S.Č.); dmitrij.kvitka@lsmu.lt (D.K.); vita.riskeviciene@lsmu.lt (V.R.); 2 Referral Animal Clinic Aisti, 01600 Vantaa, Finland; julija.tamosauskaite@gmail.com

**Keywords:** status epilepticus, cluster seizures, seizure onset, feline epilepsy

## Abstract

Epilepsy is a common neurological disorder in cats, but its seizure types, etiologies, and clinical associations remain incompletely characterized compared to dogs. This retrospective study analyzed 118 feline epilepsy cases from two veterinary hospitals in Finland and Lithuania, aiming to evaluate the distribution of seizure types and their associations with demographic, clinical, neurological, diagnostic, and diagnostic test results. Seizures were classified as single generalized (SG), cluster seizures (CS), or status epilepticus (SE). SG and CS occurred with nearly equal frequency (44.9% and 45.8%, respectively), while SE was less common (9.3%). Median age at presentation was 72 months, and no significant differences were found in age, sex, reproductive status, or body weight between seizure types. Abnormal clinical findings (*p* = 0.009) and neurological abnormalities (*p* < 0.001) were significantly more frequent in cats with SE, indicating greater neurological compromise in severe seizure forms. MRI was performed in 74.6% of cases and revealed a significant difference in utilization among seizure types (*p* = 0.004), with lower imaging rates in SE, likely reflecting clinical instability at presentation. Etiology of seizure development was established in 89 cats: idiopathic epilepsy (IE) predominated (80.9%), followed by structural (15.7%) and metabolic/toxic (3.4%) causes. Seizure type and etiology were significantly associated (*p* = 0.006), with structural epilepsy most common in SE and idiopathic epilepsy prevailing in SG and CS. Among cats with known outcomes (*n* = 35), seizure control was achieved in 68.6%, while mortality and euthanasia were higher in SE. These findings provide new insight into feline epilepsy by linking seizure type to clinical and etiological profiles, emphasizing that severe presentations are more often associated with neurological abnormalities and structural causes, whereas demographic factors alone do not predict seizure severity.

## Simple Summary

Epilepsy is among the most frequently diagnosed neurological disorders in cats. Seizures can appear as single events, come in clusters (several in a short time), or last for a very long time (status epilepticus). Cluster seizures and status epilepticus are emergencies and may require intensive treatment. We reviewed medical records of 118 cats with epileptic seizures from two veterinary hospitals in Finland and Lithuania. For each cat, we recorded age, sex, body weight, how the seizures started and progressed, clinical and neurological exam findings, blood tests, brain magnetic resonance imaging, and (when available) treatment outcomes. Most cats experienced single generalized or cluster seizures (approximately 45% each); status epilepticus was less common (9%). Age, sex, reproductive status, and body weight did not predict the seizure type. Abnormal clinical and especially neurological findings were more frequent in cats with severe seizure presentations, particularly status epilepticus. MRI was performed in three quarters of cases and helped define the cause; among cats with a known cause, idiopathic epilepsy was most common, while structural brain disease was seen mainly in cats with status epilepticus. Limited follow-up suggested seizure control was usually achievable, especially for single and cluster seizures. These results help veterinarians anticipate which cats may need urgent neurological assessment and advanced imaging while showing that routine demographics alone are poor guides to seizure severity.

## 1. Introduction

In cats, epilepsy manifests itself in various seizures with different clinical symptoms, and the main pathophysiological mechanisms are diverse and have not yet been fully investigated in veterinary neurology. Nonetheless, feline epilepsy is estimated to affect around 2% of feline population [1].

There are no current universally accepted guidelines in determination of seizure type, diagnosis and treatment in feline epilepsy, otherwise than in dogs. The International Veterinary Epilepsy Task Force (IVETF) defines canine epilepsy as a disease characterized by having at least two unprovoked epileptic seizures occurring more than 24 h apart [2]. Epileptic seizure in humans is defined by at least two unprovoked seizures occurring more than 24 h apart, the unprovoked seizure and a probability of further seizures similar to the general recurrence risk after two unprovoked seizures or a diagnosis of an epilepsy syndrome [3].

According to the IVETF, seizure types include focal seizures, generalized seizures, focal seizures evolving into generalized seizures, cluster seizures and status epilepticus [2]. Furthermore, seizure types are then further defined by etiology into idiopathic and structural epilepsy or reactive seizures [2]. Structural epilepsy in cats is defined as seizure activity resulting from an identified structural brain abnormality such as neoplasia, inflammation, infection, vascular events, congenital malformations or traumatic injury [4,5]. Differentiation between idiopathic and structural seizure disorders in cats is considered critical for establishing an accurate prognosis [4]. Nonetheless, categorizing feline epilepsy into idiopathic epilepsy and structural epilepsy remains limited and contentious [6,7]. It is important to note that, even with the use of high-field MRI, small cerebrovascular or inflammatory lesions may remain undetected, so the structural epilepsy can be challenging to be ruled out completely.

Either idiopathic epilepsy or structural epilepsy is reported as the most prevalent cause of recurrent seizures in cats, with some studies indicating structural epilepsy as the dominant etiology, while others find idiopathic epilepsy to be more common in young adult cats with normal neurological examinations [1,5,8]. Structural epilepsy was more commonly indicated than idiopathic epilepsy when status epilepticus occurred, when cats showed abnormal neurological signs between seizures, and when seizures began after the age of 7 years [5].

Structural epilepsy also indicates the presence of a brain lesion or cases where brain lesions are suspected but cannot be detected, known as cryptogenic epilepsy, as well as metabolic seizures, even if they are not classified as true epilepsy [9]. Some studies suggest that idiopathic epilepsy in cats may be more common than previously thought, challenging earlier beliefs that it was a rarer condition. Recent findings indicate that idiopathic epilepsy could have a higher prevalence in feline patients than initially recognized [6,8,10]. A substantial proportion of cats with recurrent seizures exhibit no identifiable underlying disease, suggesting a diagnosis of IE in these cases [1,4,5,8,10]. Currently, there is no genetic test available to definitively confirm idiopathic epilepsy and the diagnosis remains presumptive and is primarily established through the exclusion of other potential etiological factors [9]. Idiopathic epilepsy in cats is diagnosed using the IVETF’s tiered confidence system, Tier II being considered the clinical gold standard for most practice settings [2]. A Tier II diagnosis of IE in cats indicates that the patient has experienced at least two unprovoked epileptic seizures more than 24 h apart, demonstrates unremarkable interictal physical and neurological examinations, and has normal routine laboratory test results, with no evidence of metabolic, toxic, or reactive causes. In addition, advanced diagnostic evaluation, including MRI of the brain and cerebrospinal fluid (CSF) analysis, fails to reveal any structural brain disease or infectious/inflammatory central nervous system pathology, thereby supporting the exclusion of secondary or reactive etiologies.

Screening of the European Shorthair breed for idiopathic epilepsy in Sweden showed that the proportion of IE cases was similar to that of the general population. Therefore, this finding did not provide sufficient evidence to support a genetic basis for IE in this breed [11]. Interestingly, a recent study identified a novel pathogenic variant in the CAD gene of a Bengal kitten with intractable seizures, linking it to epileptic encephalopathy like a human genetic epilepsy syndrome which suggests that genetic mutations can contribute to epilepsy in cats [12]. An identified spontaneous model of genetic epilepsy has been discovered in laboratory cats [13]. Epileptic feline strain is valuable because it offers a naturally occurring genetic model of idiopathic epilepsy in cats, allowing researchers to investigate how inherited factors contribute to epilepsy and to observe seizure patterns comparable to those seen in other species [13].

In cats, seizures can frequently be complex focal seizures without secondary generalization [9,14]. During focal seizures, affected cats may remain motionless or display heightened ambulatory movements. Additionally focal epileptic seizures may as well generalize, leading to potential misclassification as primary generalized seizures due to the brief or subtle focal onset phase that often goes unrecognized without electroencephalographic monitoring. 

Before the large-scale study by Pakozdy [1], which found that IE accounted for only 38% of feline epilepsy cases and thus was less common than other causes, just one other recent major study had investigated the causes and classification of epilepsy in cats on a similarly large scale.

Despite extensive work on etiologic classification, the current literature largely examines how factors such as age, interictal neurological status, imaging, and biochemistry relate to etiology (idiopathic, structural, reactive), rather than to seizure type per se. Consequently, there is limited evidence on how seizure type (single generalized, cluster seizures, status epilepticus) is distributed in clinical populations and how it relates to demographic, clinical/neurological, laboratory, imaging, and outcome variables. Clarifying these seizure-type associations is necessary to inform triage, diagnostic prioritization, client counseling, and resource planning.

The primary objective was to conduct a retrospective analysis of 118 of cases of epileptic seizures in cats taken from the Referral Animal Clinic Aisti (Finland) and Dr. L. Kriaučeliūnas Small Animal Clinic at the Lithuanian University of Health Sciences (Lithuania). The aim of this study was to analyze the distribution of seizure types in cats, including single generalized seizures (SG), cluster seizures (CS), and status epilepticus (SE), and to investigate their associations with demographic characteristics, clinical and neurological findings, and diagnostic test results.

## 2. Materials and Methods

### 2.1. Study Design and Data Sources

This retrospective observational study analyzed feline epilepsy cases recorded between 2019 and 2022 in two veterinary centers: Referral Animal Clinic Aisti in Vantaa (Finland) and the Dr. L. Kriaučeliūnas Small Animal Clinic of the Lithuanian University of Health Sciences in Kaunas (Lithuania). All data used in the study were extracted from the digital medical record systems routinely employed in both institutions.

### 2.2. Case Identification and Record Screening

A predefined data retrieval strategy was implemented before initiating the study. Electronic records were searched using a list of seizure-related keywords, including: “epileptic seizures,” “epilepsy,” “cluster seizures,” “status epilepticus,” “convulsions,” “loss of consciousness,” and “involuntary episodes.”

Search results were subsequently reviewed manually. Only those entries that clearly described seizure-like events were retained for further evaluation.

### 2.3. Inclusion Criteria

Cases were eligible for analysis if the following information was fully available and traceable:

- unique patient identifier,
- sex and reproductive status,
- breed,
- birth date,
- body weight,
- date of presentation,
- detailed seizure history.

The medical history had to include information on seizure onset, recurrence, and progression, enabling classification of the initial seizure event. Cats could be either first-time presenters (no previous anti-seizure treatment) or patients already receiving anti-seizure medications.

To be included in the final dataset, each cat had to be assignable—without ambiguity—to one of the seizure type categories: single generalized (SG), cluster seizures (CS), or status epilepticus (SE). Classification followed the International Veterinary Epilepsy Task Force (IVETF) definitions [2].

### 2.4. Exclusion Criteria

Records were excluded if essential demographic data (age, sex, weight, or breed) were missing, or if the clinical description did not allow reliable identification of the seizure type.

Cases were also removed when episodes were determined to be non-epileptic (e.g., paroxysmal dyskinesia, syncope, narcolepsy, cataplexy). Poorly documented cases—lacking sufficient information for seizure onset characterization—were similarly excluded.

### 2.5. Clinical, Laboratory and Neurological Data Extraction

For each case, clinical examination findings (normal vs. abnormal), blood test results, and neurological assessments were recorded.

Magnetic resonance imaging (MRI) results were documented when available. Cats that did not undergo MRI were assigned to the “unknown etiology” group for etiological comparisons.

Seizure etiologies were categorized using the VITAMIN D framework (vascular, inflammatory, traumatic, anomalous, metabolic/toxic, idiopathic, neoplastic, degenerative).

### 2.6. Body Weight Categorization

To explore potential associations between body weight and seizure type, cats were divided into two weight groups:

- ≤5 kg, representing the majority of typical domestic cats;
- >5 kg, a threshold chosen to separate heavier or large-breed individuals.

### 2.7. Statistical Analysis

Descriptive and inferential statistics were performed using Microsoft^®^ Excel 2019 and IBM SPSS Statistics^®^ 29 (2002).

Normality of data distribution was assessed using the Kolmogorov–Smirnov test. Normally distributed variables were summarized as mean ± standard deviation (SD), while skewed or non-parametric data were presented as medians with ranges and interquartile intervals (Q1–Q3).

Comparisons between groups were conducted using Chi-square tests for categorical variables and Mann–Whitney U tests for non-parametric continuous variables. A 95% confidence level was applied, and results were considered statistically significant at *p* < 0.05.

### 2.8. Ethical Approval

The study protocol received approval from the Veterinary Section of the Bioethics Centre of the Lithuanian University of Health Sciences (No. 2024-BEC3-T-001).

### 2.9. Use of Generative Artificial Intelligence

AI-based tools (ChatGPT, GPT-5, OpenAI, San Francisco, CA, USA) were used solely to assist with English language editing, paragraph organization, and figure/table formatting. All scientific interpretations, analyses, and conclusions were made exclusively by the authors, who verified and approved the final manuscript.

## 3. Results

### 3.1. Seizure Type Distribution

In the present cohort of 118 feline epilepsy cases, the distribution of seizure types demonstrated that single generalized (SG) seizures and cluster seizures (CS) occurred with nearly equal frequency, while status epilepticus (SE) was comparatively uncommon (Table 1). SG seizures were identified in 44.9% of cats, CS in 45.8% and SE in 9.3%. Together, SG and CS accounted for over 90% of all seizure events recorded.

The relatively narrow confidence intervals for SG and CS confirm the robustness of these estimates, whereas the wider interval observed for SE reflects the smaller number of cases and the variability inherent to this severe seizure type. Chi-square goodness-of-fit testing further demonstrated that the observed seizure distribution significantly differed from an equal distribution across groups (χ^2^ = 30.63, df = 2, *p* < 0.001). Residual analysis revealed that both SG (+13.7) and CS (+14.7) occurred more frequently than expected, while SE (–28.3) was markedly underrepresented.

### 3.2. Age Distribution

**Figure 1:**
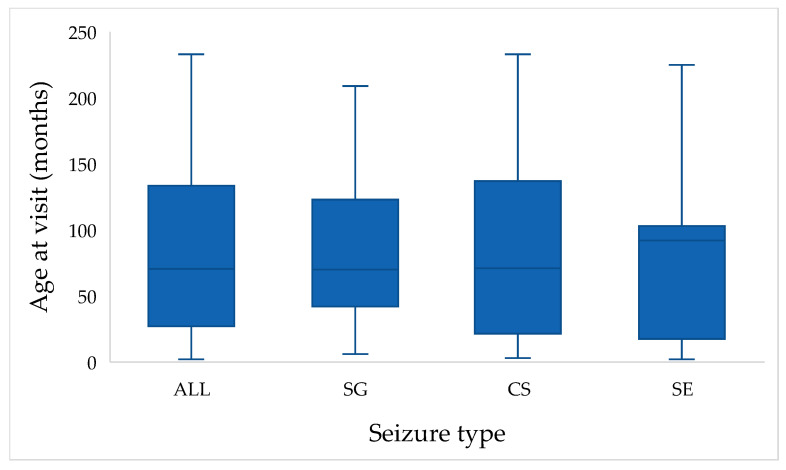
Distribution of age at visit (in months) across the study population. Boxplots show the distribution of age at presentation in cats with single generalized seizures (SG), cluster seizures (CS), and status epilepticus (SE). The “ALL” category illustrates the overall age distribution of the entire study population (*n* = 118).

The variable age at visit (in months) was available for all cats (*n* = 118). Tests of normality demonstrated that age was not normally distributed. The Kolmogorov–Smirnov test indicated a significant deviation from normality (D = 0.120, df = 118, *p* < 0.001), which was confirmed by the Shapiro–Wilk test (W = 0.923, df = 118, *p* < 0.001). The median age at visit was 72 months (Q1 = 48, Q3 = 108), with a range from 6 to 204 months. Consequently, non-parametric statistical methods were applied for further analysis of age-related variables (Figure 1).

When stratified by seizure type, median age were 70 months for SG (Q1 = 42, Q3 = 123), 71 months for CS (Q1 = 21, Q3 = 138), and 92 months for SE (Q1 = 17.5, Q3 = 103). Despite these apparent differences, the Kruskal–Wallis test revealed no statistically significant differences among seizure type groups (H = 0.578, df = 2, *p* = 0.749). Mean ranks were 61.64 for SG, 58.61 for CS, and 53.55 for SE, indicating broadly similar age distributions across seizure types (Figure 1).

### 3.3. Sex and Reproductive Status

Of the 118 cats included in the study, 55 (46.6%) were male and 63 (53.4%) were female. The sex distribution did not differ significantly from an equal ratio (χ^2^ = 0.542, df = 1, *p* = 0.461).

Age at visit was compared between males and females. The median age of males was 58 months (Q1 = 20, Q3 = 119), while that of females was 73 months (Q1 = 40, Q3 = 151.5). Although females tended to be older at presentation, the difference between sexes was not statistically significant (Mann–Whitney U = 1441.5, Z = −1.57, *p* = 0.116). Overall, age distributions in both sexes were broadly overlapping.

Reproductive status was recorded for all 118 cats included in the study. Overall, the majority of cats were neutered or spayed (85.6%, *n* = 101), while only 14.4% (*n* = 17) remained sexually intact. This distribution differed significantly from an equal proportion of intact versus neutered/spayed cats (χ^2^ = 59.80, df = 1, *p* < 0.001;). Among males, 10.9% (*n* = 6) were intact and 89.1% (*n* = 49) neutered, whereas among females, 17.5% (*n* = 11) were intact and 82.5% (*n* = 52) spayed. Statistical analysis revealed no significant difference in reproductive status distribution between sexes (χ^2^ = 0.560, df = 1, *p* = 0.454; Table 2).

When comparing age at visit between groups, intact cats had a median age of 24 months (Q1 = 8, Q3 = 70), while neutered/spayed cats were older, with a median of 76 months (Q1 = 38, Q3 = 138). Mann–Whitney U test confirmed that neutered/spayed cats presented at a significantly older age compared to intact cats (U = 490.0, Z = −2.82, *p* = 0.005). These findings suggest a potential relationship between reproductive status and the timing of epilepsy onset or clinical presentation.

Analysis of the association between reproductive status and seizure type revealed no statistically significant relationship (χ^2^ = 1.97, df = 2, *p* = 0.373; Table 3). Among cats with single generalized seizures, 84.9% (*n* = 45) were neutered and 15.1% (*n* = 8) were sexually intact. Similarly, in cluster seizure cases, 88.9% (*n* = 48) were neutered and 11.1% (*n* = 6) were intact. In the status epilepticus group, neutered animals accounted for 72.7% (*n* = 8), while intact individuals comprised 27.3% (*n* = 3). Although descriptively neutered cats were more frequent across all seizure types, the distribution did not differ significantly between groups.

When seizure types were dichotomized into SG seizures versus more severe forms (CS and SE combined), the distribution of reproductive status remained nearly identical between groups. Among cats with SG, 15.1% (*n* = 8) were sexually intact and 84.9% (*n* = 45) were neutered, while in the CS + SE group 15.8% (*n* = 9) were intact and 84.2% (*n* = 56) neutered. Chi-square analysis confirmed that these proportions did not differ significantly (χ^2^ = 0.009, df = 1, *p* = 0.926). However, reproductive status did not show any statistically significant association with the complexity of seizure type in the studied feline population.

### 3.4. Weight Distribution

Body weight among the study population (*n* = 118) ranged from 0.54 to 9.8 kg, with a median of 4.0 kg (Q1 = 3.5, Q3 = 4.95). Tests of normality indicated that weight was not normally distributed (Kolmogorov–Smirnov *D* = 0.150, *p* < 0.001; Shapiro–Wilk *W* = 0.926, *p* < 0.001). When stratified by seizure type, median body weights were 4.0 kg (Q1 = 3.7–4.95) in the SG group, 4.0 kg (Q1 = 3.5–5.0) in the CS group, and 3.5 kg (Q1 = 2.95–4.35) among cats with SE. Although cats experiencing SE tended to have slightly lower body weights, these differences were not statistically significant (Kruskal–Wallis H = 3.35, df = 2, *p* = 0.187; Table 4).

Cats were divided into two weight categories: <5 kg (*n* = 92; 78.0%) and ≥5 kg (*n* = 26; 22.0%). The distribution of seizure types did not differ significantly between these weight groups (χ^2^ = 0.28, df = 2, *p* = 0.871; Table 5).

### 3.5. Breed Distribution

Most of the cats in the study population were of mixed breed (*n* = 96, 81.4%). Purebred cats accounted for 22 cases (18.6%) and included 13 different breeds, each represented by one to four individuals. The most frequent purebred cats were Burmese (*n* = 4, 3.4%), British Shorthair (*n* = 3, 2.5%), and Maine Coon (*n* = 3, 2.5%). When stratified by seizure type, mixed-breed cats were distributed as follows: 42.7% with single generalized seizures, 49.0% with cluster seizures, and 8.3% with status epilepticus. No clear seizure-type predisposition was observed among purebred cats, as numbers within each breed were too small for statistical comparison (Table 6).

### 3.6. Seizure Onset and Clinical Presentation

**Figure 2:**
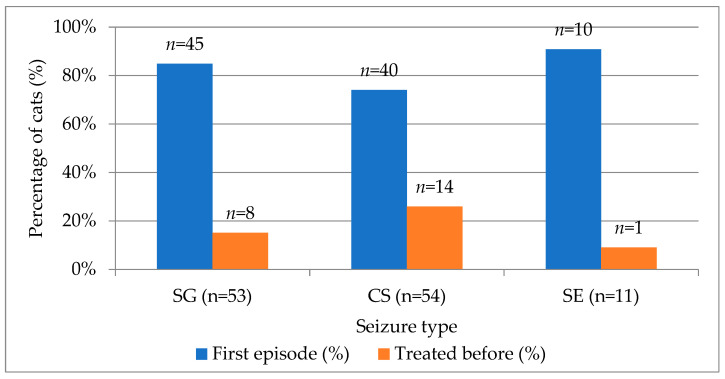
Distribution of first-episode and previously treated cats across SG, CS, SE seizure types. SG—single generalized, CS—cluster seizures, SE—status epilepticus.

At the time of their initial clinical evaluation, most cats (80.5%) were presented for their first documented epileptic episode, whereas 19.5% had received anti-seizure treatment prior to the visit. When stratified by seizure type, 84.9% of SG, 74.1% of CS, and 90.9% of SE cases represented first-episode presentations. Conversely, cats previously treated for seizures accounted for 15.1% of SG, 25.9% of CS, and 9.1% of SE cases (Figure 2). Although a higher proportion of cats with cluster seizures had a history of prior treatment, this difference did not reach statistical significance (χ^2^ = 2.84, df = 2, *p* = 0.242).

Of the 118 cats included, seizure onset patterns varied widely across seizure type categories (χ^2^ = 98.42, df = 6, *p* < 0.001; Cramer’s V = 0.646), confirming a statistically significant association between the initial seizure manifestation and the final seizure classification.

Most cats (80.5%) experienced generalized seizures from the outset, whereas 19.5% of cats had focal onset seizures. Among those cats with focal seizures at onset (*n* = 23), 55.6% progressed to cluster seizures (CS) and 44.4% to single generalized (SG) seizures.

The cats whose seizures started as single generalized episodes primarily remained within the SG category (43.5%), although some developed CS (36.7%) or SE (19.7%). In contrast, cats with cluster seizure onset typically remained within the CS group (73.2%), while 26.8% advanced to SE (Table 7). All cases initially presenting as status epilepticus (SE) maintained this classification.

#### 3.6.1. Blood Test Findings

Blood test results were available for all 118 cats included in the study. Across all seizure-type categories presented in Table 1, a total of 80 cats (67.8%) had completely normal hematologic and biochemical values, whereas 38 cats (32.2%) showed at least one abnormality, most commonly mild hyperglycemia, hypokalemia, anemia, or moderate increases in renal or liver parameters. Other sporadic findings included hyperglobulinemia and hypercholesterolemia (Table S1). When stratified by seizure type, normal blood results were recorded in 71.7% of cats with SG seizures, 66.7% of those with CS, and 54.5% of those with status SE. Conversely, abnormal findings were more frequent among cats experiencing SE (45.5%) than among those with SG (28.3%) or CS (33.3%) seizures. However, the differences across seizure type groups were not statistically significant (χ^2^ = 1.29, df = 2, *p* = 0.526).

#### 3.6.2. Clinical Examination Findings

Clinical examination findings were available for all 118 cats.

Overall, 110 cats (93.2%) showed normal findings during clinical evaluation, while 8 cats (6.8%) exhibited abnormalities. When stratified by seizure type, normal examination results were observed in 92.5% of cats with single generalized seizures, 98.1% of those with cluster seizures, and 72.7% of those with status epilepticus. Conversely, abnormal findings were more frequent in cats presenting with SE (27.3%) compared to SG (7.5%) and CS (1.9%) groups. A statistically significant association was detected between seizure type and clinical examination status (χ^2^ = 9.43, df = 2, *p* = 0.009).

#### 3.6.3. Neurological Examination Findings

Neurological examination findings were available for all 118 cats. Overall, 62 cats (52.5%) demonstrated abnormal neurological findings, while 56 cats (47.5%) had normal examinations. When evaluated across seizure type groups, abnormal neurological findings were detected in 37.7% of cats with SG seizures, 57.4% of those with CS, and in all cats (100%) presenting with SE. Conversely, normal neurological examinations were most common among SG (62.3%) and CS (42.6%) cats, whereas none of the SE cats exhibited normal findings. A statistically significant association was found between seizure type and neurological status (χ^2^ = 15.11, df = 2, *p* < 0.001).

### 3.7. Diagnostics and Etiological Classification

Magnetic resonance imaging was performed on most cats diagnosed with epileptic seizures. Out of 118 evaluated cases, MRI examinations were carried out in 88 cats (74.6%), while 30 cats (25.4%) did not undergo MRI. The distribution analysis demonstrated a statistically significant predominance of cases with performed MRI compared to those without (χ^2^ = 28.51, df = 1, *p* < 0.001).

**Figure 3:**
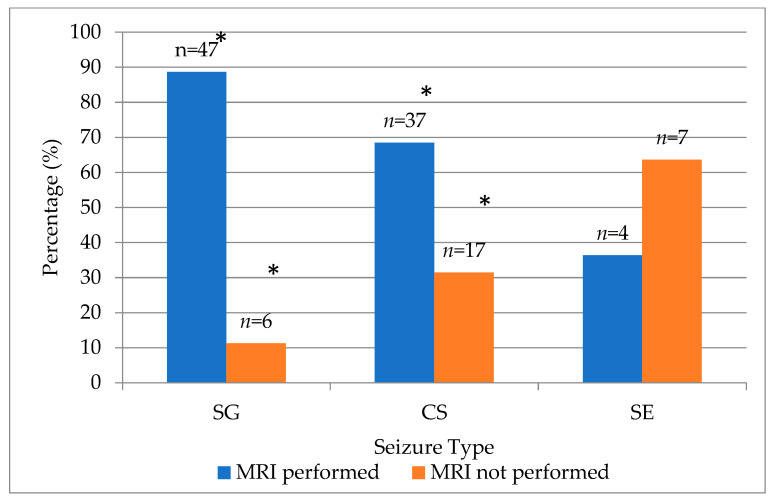
Distribution of MRI examination status across seizure type groups in cats. (* indicates significant *p* < 0.001 deviation by χ^2^ test). SG—single generalized, CS—cluster seizures, SE—status epilepticus.

When evaluated across seizure type groups, the frequency of MRI performance differed significantly (χ^2^ = 10.86, df = 2, *p* = 0.004). MRI examinations were performed in 88.7% of cats with single generalized seizures, 68.5% of those with cluster seizures and only 36.4% of those presenting with status epilepticus (Figure 3).

Although MRI examination was performed in 88 cats, etiological diagnosis was successfully established in 89 cases (75.4%). This discrepancy reflects one cat diagnosed with metabolic epilepsy based on additional diagnostic testing other than MRI. The remaining 29 cats (24.6%) were classified as having an unknown etiology due to incomplete diagnostic data. Among the identified cases, idiopathic epilepsy was the most common etiology, accounting for 80.9% (*n* = 72), followed by structural epilepsy in 15.7% (*n* = 14) and metabolic/toxic epilepsy in 3.4% (*n* = 3) (Table 8).

The median age at presentation differed slightly between etiological categories: idiopathic epilepsy—60 months (Q1 = 28.5, Q3 = 109.5), structural epilepsy—104 months (Q1 = 40, Q3 = 156), and metabolic/toxic epilepsy—55 months (Q1 = 36, Q3 = 96.5). Although cats with structural epilepsy tended to be older at presentation compared with those diagnosed with idiopathic or metabolic epilepsy, these differences were not statistically significant (Kruskal–Wallis H = 1.353, df = 2, *p* = 0.508). Mean ranks were 43.67 for idiopathic, 52.36 for structural, and 42.50 for metabolic epilepsy, indicating overlapping age distributions across diagnostic categories (Table 8).

When comparing etiological categories across seizure types, idiopathic epilepsy predominated among cats presenting with single generalized (SG) and cluster seizures (CS), accounting for 76.6% and 92.1% of these cases, respectively. In contrast, structural epilepsy was most frequently associated with status epilepticus (SE), representing 75.0% of cats within this group. Metabolic causes were rare and occurred only among cats with SG or CS seizures. Statistical analysis confirmed a significant association between seizure type and etiological category (χ^2^ = 14.48, df = 4, *p* = 0.006) (Table 9).

Based on the VITAMIN-D classification, idiopathic epilepsy was the most common diagnosis, accounting for 61.0% (*n* = 72) of all cases and 80.9% among cats with a known etiology (Table 10). Among structural-related causes within the VITAMIN-D framework, inflammatory and neoplastic etiologies were identified in 6 cats (5.1%) each, anomalous malformations in 2 cats (1.7%), and metabolic/toxic disorders in 3 cats (2.5%). No vascular, traumatic, or degenerative causes were detected in this cohort. The etiology remained unknown in 29 cats (24.6%).

### 3.8. Outcome

Outcome data were available for 35 cats (29.7%), while 83 cases (70.3%) lacked follow-up information. Among those with known outcomes, seizure control was achieved in 68.6% of cats, 8.6% were euthanized due to poor seizure control or disease progression, 8.6% required changes in medication because of adverse effects, 8.6% died during the observation period, and 5.7% needed an increased anti-seizure medication (ASM) dosage (Table 11).

When outcomes were compared across seizure types, seizure control was recorded in 66.7% of cats with SG seizures, 77.8% with CS, and 40.0% with SE. Euthanasia occurred most frequently in the CS group (11.1%) and SE group (20.0%), while medication change due to side effects was reported only in the SG group (25.0%). Due to small subgroup sizes, no statistically significant association between seizure type and outcome was detected.

## 4. Discussion

### 4.1. Seizure Type Distribution

Seizure type distribution in this cohort was dominated by single generalized (SG; 44.9%) and cluster seizures (CS; 45.8%), with status epilepticus (SE; 9.3%) comparatively uncommon. This pattern aligns with prior feline reports in which CS were frequent and SE occur in a minority of cases [11,15]. However, our data may not reflect primary care feline population, because referral populations usually skew towards more complex seizure presentation. Hence, caution is warranted in applying these proportions to general practice settings.

### 4.2. Age Distribution

The median age at first recorded epileptic event in our cohort was 72 months, which agrees with previous reports [9,16,17]. Cases presenting with status epilepticus in our study tended to be older (median 92 months), although this difference did not reach statistical significance. Conversely, a large primary care cohort reported a higher mean age (8.9 years) at first seizure for recurrent seizure disorders [18]. This discrepancy likely reflects population differences, as our data were obtained from two referral veterinary hospitals rather than a general feline population.

There were no significant age differences between cats presenting with SG, CS or SE. To our knowledge, prior feline epilepsy studies describe the frequencies of cluster seizures and status epilepticus and summarize age distributions but do not demonstrate a consistent association between age and seizure type. Instead, most analyses evaluate association between age and etiology. Therefore, our results provide cohort level evidence that age alone is not a strong discriminator of seizure type in cats, even though advancing age is frequently linked to structural causes in the broader literature [1].

### 4.3. Sex and Reproductive Status

In the current literature, there is no strong evidence that either sex or reproductive status influences the risk of feline epilepsy. In a large primary care cohort, breed, sex, and reproductive status were not associated with recurrent seizure disorders or epilepsy after multivariable adjustment. Although, advancing age was the dominant demographic risk factor [18].

Earlier studies and etiology specific series likewise report near equal male and female distributions among cats with seizures and do not identify sex as a risk factor, which aligns with our findings [7,11]. However, most feline studies either show no association between sex/reproductive status and risk of epilepsy or simply report sex and reproductive status proportions without testing whether age at onset or initial clinical presentation (SG, CS or SE) differs by reproductive status.

Our data suggest a potential association between reproductive status and the timing of epilepsy onset and/or initial clinical presentation. Neutered/spayed cats were presented at a significantly older age than intact cats. Yet, this age difference may reflect cohort composition rather than a causal effect of reproductive status, because neutered cats are typically older and may have a different disease spectrum, underscoring the need for prospective feline studies with appropriate control for age, etiology, and referral bias.

In addition, reproductive status showed no statistically significant association with seizure type in our cohort. Prior feline studies report seizure type frequencies and signalment but do not demonstrate a reproductive status and seizure type relationship [1,11,15,18,19]. Hence, definitive assessment will depend on well powered prospective studies that account for key confounders, particularly age and cause of seizures.

### 4.4. Weight Distribution

Current literature suggests that body weight is not associated with recurrent seizure disorders or epilepsy risk in cats [18].

In our cohort, body weight did not differ significantly across seizure types, indicating no observable association between weight and seizure presentation. Collectively, available data and our results indicate that body weight does not influence seizure phenotype in cats. Accordingly, risk assessment should prioritize factors with stronger signal, such as age at onset or etiologic category.

### 4.5. Breed Distribution

In this cohort, mixed breed cats comprised the majority (81.4%), whereas purebreds accounted for 18.6%, spanning 13 breeds, each represented by one to four individuals. This distribution is consistent with demographic patterns reported in seizure affected feline populations [1,18].

Within the mixed breed subset, seizure phenotypes were broadly represented: single generalized (SG) seizures 42.7%, cluster seizures (CS) 49.0%, and status epilepticus (SE) 8.3%. Although Burmese (*n* = 4), British Shorthair (*n* = 3), and Maine Coon (*n* = 3) were the most frequently represented purebreds, breed specific sample sizes were insufficient to permit robust comparative analyses. Nevertheless, our results do not support a link between breed and seizure complexity, although larger studies are needed to confirm this.

### 4.6. Seizure Onset and Clinical Presentation

Prior reports [9,20] indicate that seizure semiology can evolve rapidly to more severe phenotype. Therefore, the initial manifestation may be overlooked at presentation. However, to our knowledge, there are no feline studies that quantitatively characterize longitudinal progression between seizure types.

In our cohort, initial seizure phenotype was significantly associated with the final seizure classification. Among cats presenting initially with focal seizures, 55.6% subsequently exhibited cluster seizures (CS) and 44.4% evolved to single generalized seizures (SG). Of those with an initial SG, 36.7% later developed CS and 19.7% experienced status epilepticus (SE). Majority of cats with an initial CS (73.2%) remained in the CS category over time. Nonetheless, misclassification at onset cannot be excluded, particularly if early focal seizures were unrecognized before progression to more complex presentations. Clinically, this data underscores the importance of vigilant follow up and early intervention strategies, as initial seizure type may hold prognostic value for future complexity.

### 4.7. Blood Test Findings

No feline studies have demonstrated an association between routine blood biochemical abnormalities and seizure type. Consistent with this, no statistically significant associations were found between biochemical abnormalities and type of seizure in this study. Nevertheless, a higher proportion of cats with status epilepticus (45.5%) had at least one biochemical abnormality at presentation (e.g., mild hyperglycemia, hypokalemia, or elevated renal and liver values) compared with those with single generalized seizures (28.3%). Although we did not stratify this data by seizure etiology, this tendency is consistent with previous reports that reactive or metabolic etiologies are overrepresented among more severe seizure presentations [21].

### 4.8. Clinical and Neurological Examination Findings

Systemic and cerebral derangements including increased intracranial pressure, hyperthermia, and acid—base disturbances are well described after prolonged or repeated seizure activity [22,23]. Previous feline studies mostly relate clinical and neurological examination abnormalities to seizure etiology rather than seizure type [1,5,16,24]. In this study, severe seizure presentations (CS, SE) were significantly associated with a higher frequency of abnormal clinical findings (commonly tachycardia, abnormal respiratory patterns, or poor body condition) and neurological abnormalities (ataxia, dull mentation, or postural asymmetry) were particularly prevalent in SE. Therefore, our seizure stratified analysis adds new evidence. Furthermore, these data support prior evidence that prolonged ictal activity leads to postictal brain and systemic dysfunction, resulting in abnormal clinical and neurological findings.

### 4.9. Diagnostics and Etiological Classification

There are wide variations in MRI diagnostic usage in feline epilepsy studies. Previous studies report as little as 12–27% cases while in comparison more recent studies are commonly MRI based by design [1,5,25]. MRI utilization was high (74.6%) in our cohort, consistent with its role as the “gold standard” imaging modality for evaluating intracranial pathology in cats. However, because the cohort was drawn from two referral veterinary centers, MRI utilization could possibly be overrepresented, relative to primary care populations.

MRI utilization differed significantly by seizure type in our cohort. Cats with SE (36.4%) were significantly less (*p* < 0.005) likely to receive MRI than SG (88.7%) or CS (68.5%) cases. This pattern may reflect clinical considerations (hemodynamic instability, anesthetic risk or the need to prioritize seizure control), that may limit MRI in cats with severe seizure presentation. To our knowledge, no prior feline studies have reported MRI uptake, stratified by presenting seizure type, rendering these findings novel.

Previous studies report widely varying proportions of seizure etiologies. Notable differences occur between primary and referral populations and the extensive diagnostic workup, including MRI availability and CSF analysis. In our cohort idiopathic epilepsy (IE) was the most frequent diagnosis, identified in 80.9% of cases. This exceeds most prior reports, where IE was less frequent: 25% [1], 38% [9], and 48.9% [26]. The higher IE proportion in our series can possibly be influenced by low number of SE cats that underwent MRI diagnostic. SE appears more common with structural pathology, rather than IE [5], making this group significantly more common in our study. In addition, our cohort consisted of mostly referred cases. Therefore, many metabolic/toxic cases could have possibly be ruled out at the primary care, without MRI.

When age distributions across etiologic categories were compared, older cats were slightly more often diagnosed with structural epilepsy, but the differences were not statistically significant. This tendency aligns to other studies documenting that age differs by etiology and IE occurs in younger cats while structural and most reactive cases present in older age [1,24].

Seizure etiology and seizure type were significantly (*p* < 0.05) associated in our study. Among cats with structural epilepsy, 75.0% presented with SE, whereas idiopathic epilepsy predominated among SG (76.6%) and CS (92.1%) presentations. The overrepresentation of SE in structural epilepsy, aligns with prior report [5]. In contrast, prior studies did not identify differences in CS prevalence across etiologies [5,15], making the association with idiopathic epilepsy observed here a potentially novel finding.

### 4.10. VITAMIN D

Idiopathic epilepsy predominated in our cohort (61% of all cases) exceeding the idiopathic proportions 25% and 38% reported in previous studies [1,5], while structural causes were comparatively infrequent compared to 50 and 62% reported elsewhere [1,5]. Furthermore, low frequencies of neoplasia and inflammatory disease, each 5.1%, are in contrast with higher neoplastic and inflammatory counts in earlier cohorts, where neoplasia and hippocampal necrosis were prominent contributors to structural epilepsy [5]. The absence of vascular, traumatic, or degenerative categories in our cohort is consistent with reports that these etiologies are relatively uncommon or can be under detected without advanced imaging or further pathological diagnostics [1,6]. These discrepancies between studies likely reflect differences in study population (referral vs. general practice), diagnostic strategy, age distribution and should be interpreted with caution.

### 4.11. Outcome

Direct comparisons of outcome by SG, CS and SE in feline studies are limited. Most papers analyze outcome by etiology, rather than by seizure type. Overall, it is reported that SE carries the highest short-term risk, while CS demands close management due to recurrence risk. In contrast, SG cases are often with better overall outlook [27]. Our results suggest that seizure control were achieved in majority of CS (77.8%) and SG (66.7%) cases, while death and euthanasia was more frequent among cats presenting with SE (40.0% and 20.0%, respectively). Nevertheless, limited follow-up precluded formal statistical comparisons. Therefore, prospective studies with sufficient power are needed to determine whether seizure type is associated with outcome.

### 4.12. Limitations of the Study

This study has several limitations inherent to retrospective clinical research. Although all procedures and data collection steps were methodically planned to follow a unified approach, the dataset was obtained from two veterinary clinics, which may still introduce variability in diagnostic practices, completeness of medical records, and consistency of follow-up. Additionally, some etiological subgroups contained relatively few cases, which may limit the strength of within-group comparisons; however, these small numbers did not distort the overall conceptual framework or the broader conclusions of the study. Despite these factors, the study provides a valuable overview of seizure type distribution and associated clinical features in cats, offering clinically meaningful insights for veterinary practitioners.

## 5. Conclusions

This study provides one of the first systematically structured comparisons of seizure types in cats—single generalized seizures, cluster seizures, and status epilepticus—evaluated across demographic factors, clinical and neurological findings, diagnostic test results, and outcomes. By focusing on seizure type rather than solely on etiology, this work addresses an under-explored area in feline epilepsy research and offers new clinically applicable insights for diagnostic prioritization and prognosis.

This study demonstrated that single generalized and cluster seizures were the most common seizure types in the investigated cat population, while status epilepticus was relatively rare but associated with abnormal clinical and neurological findings, structural brain lesions, and poorer outcomes. Idiopathic epilepsy predominated overall, representing the majority of cases with a known etiology, whereas structural causes were primarily linked to status epilepticus and older age at presentation. No significant associations were found between seizure type and sex, reproductive status, or body weight, indicating that demographic factors do not predict seizure severity in cats. Seizure progression from focal or single generalized forms to cluster seizures or status epilepticus was observed, suggesting that epileptic activity in cats can evolve over time. These findings emphasize the need for comprehensive diagnostic evaluation—including MRI and neurological examination—in all feline epilepsy cases, especially those presenting with severe or progressive seizure patterns. Early recognition and individualized management may improve outcomes and help refine prognostic indicators in feline epilepsy.

## Figures and Tables

**Table 1:** Distribution of seizure types in cats with epilepsy (*n* = 118).

Seizure Type	Observed *n*	% (95% CI)	Expected *n*	Residual
SG	53	44.9 (36.4–54.2)	39.3	+13.7
CS	54	45.8 (36.5–54.2)	39.3	+14.7
SE	11	9.3 (4.3–14.4)	39.3	–28.3
Total	118	100	–	–

SG—single generalized, CS—cluster seizures, SE—status epilepticus.

**Table 2:** Distribution of reproductive status between study sites.

Sex	SI, *n* (%)	NEU/SPD, *n* (%)	Total, *n* (%)	χ^2^; *p*-Value
Males	6 (10.9%)	49 (89.1%)	55 (100%)	
Females	11 (17.5%)	52 (82.5%)	63 (100%)	
Total	17 (14.4%)	101 (85.6%)	118 (100%)	χ^2^ = 0.560; *p* = 0.454

SI—sexually intact; NEU—neutered; SPD—spayed.

**Table 3:** Distribution of seizure types according to reproductive status.

Seizure Type	SI, *n* (%)	NEU/SPD, *n* (%)	χ^2^; *p*
SG (*n* = 53)	8 (15.1%)	45 (84.9%)	χ^2^ = 1.97; *p* = 0.373
CS (*n* = 54)	6 (11.1%)	48 (88.9%)	
SE (*n* = 11)	3 (27.3%)	8 (72.7%)	
Total (*n* = 118)	17 (14.4%)	101 (85.6%)	

SG—single generalized, CS—cluster seizures, SE—status epilepticus. SI—sexually intact; NEU—neutered, SPD—spayed.

**Table 4:** Distribution of seizure types according to the cat weight.

Seizure Type	*n*	Median Weight (kg)	Q1–Q3	Min–Max	Kruskal–Wallis
SG	53	4.00	3.5–4.95	2.40–9.80	H = 3.35/*p* = 0.187
CS	54	4.00	3.50–5.00	1.70–7.40	
SE	11	3.50	3.00–4.90	0.54–5.80	

SG—single generalized, CS—cluster seizures, SE—status epilepticus.

**Table 5:** Distribution of seizure types according to weight groups (<5 kg vs. ≥5 kg).

Weight Group	SG (*n* = 53)	CS (*n* = 54)	SE (*n* = 11)	Total (*n* = 118)	χ^2^/*p*
<5 kg	42 (79.2%)	41 (75.9%)	9 (81.8%)	92 (100%)	χ^2^ = 0.28/*p* = 0.871
≥5 kg	11 (20.8%)	13 (24.1%)	2 (18.2%)	26 (100%)	

SG—single generalized, CS—cluster seizures, SE—status epilepticus.

**Table 6:** Distribution of cat breeds according to seizure type.

Breed	SG (*n* = 53)	CS (*n* = 54)	SE (*n* = 11)	Total (*n* = 118)
Mixed breed	41 (42.7%)	47 (49.0%)	8 (8.3%)	96 (81.4%)
British Shorthair	2 (66.7%)	1 (33.3%)	0 (0.0%)	3 (2.5%)
Burmese	2 (50.0%)	1 (25.0%)	1 (25.0%)	4 (3.4%)
Maine Coon	2 (66.7%)	0 (0.0%)	1 (33.3%)	3 (2.5%)
Other breeds *	6 (54.5%)	4 (36.4%)	1 (9.1%)	11 (9.3%)
Total	53	54	11	118 (100%)

* Includes Devon Rex, Manx, Norwegian Forest Cat, Ocicat, Persian, Ragdoll, Russian Blue, Sphynx, Siamese, Siberian Cat. SG—single generalized, CS—cluster seizures, SE—status epilepticus.

**Table 7:** Seizure onset and progression patterns in cats.

Started as	Progressed to	%
Focal seizures	CS/SG	55.6%/44.4%
Single generalized	Remained SG → CS → SE	43.5% → 36.7% → 19.7%
Cluster seizures	Remained CS → SE	73.2% → 26.8%
Status epilepticus	Remained SE	100.0%

SG—single generalized, CS—cluster seizures, SE—status epilepticus.

**Table 8:** Etiological classification of feline epilepsy cases with corresponding age distribution.

Etiological Category	*n* (%)	Median Age (Months)	Q1	Q3	Mean Rank
Idiopathic epilepsy	72 (80.9%)	60	28.5	109.5	43.67
Structural epilepsy	14 (15.7%)	104	40	156	52.36
Metabolic epilepsy	3 (3.4%)	55	36	96.5	42.50
Total	89 (100%)	—	—	—	—

**Table 9:** Distribution of etiological categories across seizure types in cats with epilepsy.

Etiological Category	SG (*n* = 47)	CS (*n* = 38) *	SE (*n* = 4) *	Total (*n* = 89)
Idiopathic	36 (76.6%)	35 (92.1%) †	1 (25.0%) ‡	72 (80.9%)
Structural	9 (19.1%)	2 (5.3%) †	3 (75.0%) ‡	14 (15.7%)
Metabolic	2 (4.3%)	1 (2.6%)	0 (0.0%)	3 (3.4%)
Total	47 (52.8%)	38 (42.7%)	4 (4.5%)	89 (100%)

* Significant overall association between seizure type and etiological category (*p* = 0.006). † Proportions within the CS group differ significantly from SE (*p* < 0.05). ‡ Proportions within the SE group differ significantly from both SG and CS (*p* < 0.05). SG—single generalized, CS—cluster seizures, SE—status epilepticus.

**Table 10:** Distribution of etiological categories according to the VITAMIN-D classification in cats.

VITAMIN-D Category	*n* (of 118)	% of Total	*n* (of 89 with Known Etiology)	% of Known
V—Vascular	0	0.0	0	0.0
I—Infectious/Inflammatory	6	5.1	6	6.7
T—Traumatic	0	0.0	0	0.0
A—Anomalous	2	1.7	2	2.2
M—Metabolic/Toxic	3	2.5	3	3.4
I—Idiopathic	72	61.0	72	80.9
N—Neoplastic	6	5.1	6	6.7
D—Degenerative	0	0.0	0	0.0
Unknown	29	24.6	—	—

**Table 11:** Treatment outcomes in cats with epilepsy (*n* = 35) and their distribution across seizure types.

Outcome	SG (*n* = 12)	CS (*n* = 18)	SE (*n* = 5)	Total (*n* = 35)
Euthanasia	0 (0.0%)	2 (66.7%)	1 (33.3%)	3 (8.6%)
Increased ASMs	0 (0.0%)	2 (100.0%)	0 (0.0%)	2 (5.7%)
Controlled	8 (33.3%)	14 (58.3%)	2 (8.3%)	24 (68.6%)
Death	1 (33.3%)	0 (0.0%)	2 (66.7%)	3 (8.6%)
Medication changed due to side effects	3 (100.0%)	0 (0.0%)	0 (0.0%)	3 (8.6%)
Total	12 (34.3%)	18 (51.4%)	5 (14.3%)	35 (100%)

SG—single generalized, CS—cluster seizures, SE—status epilepticus.

## Data Availability

The data presented in this study are available on request from the corresponding author. The data are not publicly available due to privacy or ethical restrictions.

## Supplementary Materials

The following supporting information can be downloaded at https://www.mdpi.com/article/10.3390/ani15233497/s1, Table S1: Descriptive statistics of abnormal hematologic parameters by seizure type.

## Abbreviations

The following abbreviations are used in this manuscript:

ASMs	Anti-seizure medications
CI	Confidence Interval
CS	Cluster Seizures
CSF	Cerebrospinal fluid
EEG	Electroencephalography
IE	Idiopathic Epilepsy
IVETF	International Veterinary Epilepsy Task Force
MRI	Magnetic Resonance Imaging
NEU	Neutered
Q1, Q3	First and Third Quartile
SD	Standard Deviation
SE	Status Epilepticus
SG	Single Generalized (seizure)
SI	Sexually Intact
SPD	Spayed
VITAMIN D	Vascular, Inflammatory, Trauma, Anomaly, Metabolic/Toxic, Idiopathic, Neoplastic, Degenerative
