# Supplementary material for: Clinical and Diagnostic Features of Feline Epilepsy: Distribution of Seizure Types and Associated Factors
Source: Animals (Basel). 2025 Dec 4;15(23):3497. doi: 10.3390/ani15233497 (PMC12691115; doi:10.3390/ani15233497)
Supplement: Supplementary file 1 [file animals-15-03497-s001.zip › animals-3999114-supplementary.pdf]

Table S1. Descriptive statistics of abnormal hematologic parameters by seizure type.

| Abnormal hematologic parameters | Seizures group | n | Mean (SD) *or Actual value (a. v.)* | Min – Max | Median |
|---|---|---|---|---|---|
| RBC (×10⁶/μL) | SG | 3 | 4.45 (0.10) | 4.35 – 4.55 | 4.44 |
| | CS | 2 | *4.46, 4.50 (a. v.)* | – | – |
| | SE | 2 | *4.35, 14.90 (a. v.)* | – | – |
| HCT (%) | SG | 1 | *25.0 (a. v.)* | – | – |
| | CS | 1 | *26.0 (a. v.)* | – | – |
| | SE | 1 | *66.0 (a. v.)* | – | – |
| HGB (g/dL) | SG | 1 | *80.0 (a. v.)* | – | – |
| | CS | 0 | – | – | – |
| | SE | 1 | *20.0 (a. v.)* | – | – |
| WBC (×10⁹/L) | SG | 2 | *5.40, 21.50 (a. v.)* | – | – |
| | CS | 1 | *4.90 (a. v.)* | – | – |
| | SE | 1 | *20.50 (a. v.)* | – | – |
| PLT (×10³/μL) | SG | 1 | *89.0 (a. v.)* | – | – |
| | CS | 1 | *90.0 (a. v.)* | – | – |
| | SE | 0 | – | – | – |
| GLU (mmol/L) | SG | 7 | 8.84 (0.18) | 8.60 – 9.10 | 8.90 |
| | CS | 6 | 7.48 (2.00) | 3.40 – 8.80 | 8.70 |
| | SE | 4 | 12.75 (4.30) | 8.70 – 17.76 | 9.40 |
| K (mmol/L) | SG | 6 | 3.38 (0.12) | 3.30 – 3.60 | 3.30 |
| | CS | 5 | 3.46 (0.12) | 3.30 – 3.60 | 3.40 |
| | SE | 2 | *3.30, 3.30 (a. v.)* | – | – |
| CREA (μmol/L) | SG | 4 | 181.50 (72.5) | 67.0 – 231.0 | 214.0 |
| | CS | 3 | 251.0 (63.0) | 201.0 – 326.0 | 228.0 |
| | SE | 1 | *223.0 (a. v.)* | – | – |
| UREA (mmol/L) | SG | 4 | 12.55 (0.96) | 11.20 – 13.50 | 12.35 |
| | CS | 4 | 20.93 (14.70) | 11.20 – 44.60 | 13.4 |
| | SE | 1 | *11.30 (a. v.)* | – | – |
| SDMA (μg/dL) | SG | 2 | *17.0, 27.0 (a. v.)* | – | – |
| | CS | 0 | – | – | – |
| | SE | 0 | – | – | – |
| ALB (g/L) | SG | 0 | – | – | – |
| | CS | 1 | *50.0 (a. v.)* | – | – |
| | SE | 0 | – | – | – |
| GLOB (g/L) | SG | 1 | *58.0 (a. v.)* | – | – |
| | CS | 0 | – | – | – |
| | SE | 1 | *63.0 (a. v.)* | – | – |
| ALT (U/L) | SG | 1 | *168.0 (a. v.)* | – | – |
| | CS | 1 | *1000.0 (a. v.)* | – | – |
| | SE | 0 | | – | – |
| ALP (U/L) | SG | 0 | | – | – |
| | CS | 1 | *208.0 (a. v.)* | – | – |
| | SE | 0 | – | – | – |
| GGT (U/L) | SG | 0 | – | – | – |
| | CS | 1 | *10.0 (a. v.)* | – | – |

| | | | | | |
|---|---|---|---|---|---|
| | SE | 0 | – | – | – |
| Ca (mmol/L) | SG | 0 | – | – | – |
| | CS | 1 | *1.90 (a. v.)* | – | – |
| | SE | 0 | – | – | – |
| Chol (mg/dL) | SG | 0 | – | – | – |
| | CS | 2 | *187.0, 187.0 (a. v.)* | – | – |
| | SE | 0 | – | – | – |
| PHB (μg/mL) | SG | 0 | – | – | – |
| | CS | 2 | *7.0, 60.0 (a. v.)* | – | – |
| | SE | 0 | – | | – |

a. v. – Actual value is provided when there were only 1 or 2 cases; SD – Standard deviation.

SG – single generalized, CS – cluster seizures, SE – status epilepticus. RBC – Red blood cells; HCT – Hematocrit; HGB – Hemoglobin; WBC – White blood cells; PLT – Platelets; GLU – Glucose; K – Potassium; CREA – Creatinine; UREA – Blood urea nitrogen; SDMA – Symmetric dimethylarginine; ALB – Albumin; GLOB – Globulins; ALT – Alanine aminotransferase; ALP – Alkaline phosphatase; GGT – Gamma-Glutamyl transferase; Ca – Calcium; Chol – Cholesterol; PHB – Phenobarbital (Serum level).
